# Hematopoietic mosaic chromosomal alterations in the New England Centenarian Study.

**DOI:** 10.1093/geroni/igab046.2544

**Published:** 2021-12-17

**Authors:** Anastasia Leshchyk, Giulio Genovese, Stefano Monti, Thomas Perls, Paola Sebastiani

**Affiliations:** 1 Boston University, Brookline, Massachusetts, United States; 2 Broad Institute, Boston, Massachusetts, United States; 3 Boston University, Boston, Massachusetts, United States; 4 Tufts Medical Center, Physician Organization, BOSTON, Massachusetts, United States

## Abstract

Mosaic chromosomal alterations (mCAs) are structural alterations that include deletions, duplications, or copy-neutral loss of heterozygosity. mCAs are reported to be associated with survival, age, cancer, and cardiovascular disease. Previous studies of mCAs in large population-based cohorts (UK Biobank, MGBB, BioBank Japan, and FinnGen) have demonstrated a steady increase of mCAs as people age. The distribution of mCAs in centenarians and their offspring is not well characterized. We applied MOsaic CHromosomal Alteration (MoChA) caller on 2298 genome-wide genotype samples of 1582 centenarians, 443 centenarians’ offspring, and 273 unrelated controls from the New England Centenarian Study (NECS). Integrating Log R ratio and B-allele frequency (BAF) intensities with genotype phase information, MoChA employs a Hidden Markov Model to detect mCA-induced deviations in allelic balance at heterozygous sites consistent with genotype phase in the DNA microarray data. We analyzed mCAs spanning over 100 k base pairs, with an estimated cell fraction less than 50%, within samples with genome-wide BAF phase concordance across phased heterozygous sites less than 0.51, and with LOD score of more than 10 for the model based on BAF and genotype phase. Our analysis showed that somatic mCAs increase with older age up to approximately 102 years, but the prevalence of the subjects with mCAs tend to decrease after that age, thus suggesting that accumulation of mCAs is less prevalent in long-lived individuals. We also used Poisson regression to show that centenarians and their offspring tend to accumulate less mCA (RR = 0.63, p=0.045) compared to the controls.

